# Comparison of Cranial Cruciate Ligament Rupture Incidence among Parous and Nulliparous Rottweiler Bitches: Evidence from a Lifetime Cohort Study Supporting a Paradigm of Pregnancy-Associated Protection against Subsequent Non-Reproductive Disease Outcomes

**DOI:** 10.3390/ani14172608

**Published:** 2024-09-07

**Authors:** Andres E. Carrillo, Aimee H. Maras, Cheri L. Suckow, Emily C. Chiang, David J. Waters

**Affiliations:** 1 Department of Exercise Science, School of Health Sciences, Chatham University, Pittsburgh, PA 15232, USA; acarrillo@chatham.edu; 2 Center for Exceptional Longevity Studies, Gerald P. Murphy Cancer Foundation, West Lafayette, IN 47906, USA; ahmaras@aol.com (A.H.M.); cheri@gpmcf.org (C.L.S.); emilychiang1225@gmail.com (E.C.C.); 3 Department of Veterinary Clinical Sciences and the Center on Aging and the Life Course, Purdue University, West Lafayette, IN 47907, USA

**Keywords:** dog, epigenetics, reproduction, ACL rupture, cruciate ligament disease, parity, longevity, risk factors, risk reduction, ovariohysterectomy

## Abstract

**Simple Summary:**

For more than a decade, researchers have been gathering evidence suggesting pregnancy may alter the resistance of women to the development of diseases later in life. We wondered if parity—pregnancy resulting in live offspring—could enhance or diminish resistance of bitches to subsequent non-reproductive disease conditions, a hypothesis previously untested in domestic dogs. To pursue this goal, we constructed detailed lifetime reproductive and medical histories from 65 purebred Rottweiler bitches enrolled in an exceptional longevity study. An advantage of studying these bitches was that we could conduct a long-term evaluation of the relationship between parity and the lifelong occurrence of a commonly diagnosed degenerative disease, cranial cruciate ligament (CCL) rupture. Notably, we found parous bitches had a significantly lower likelihood of CCL rupture than nulliparous bitches. This 5.5-fold lower incidence of CCL rupture among parous females could not be explained by differences in diet, physical activity, overweight body condition, or reason for nulliparity. Our work may guide future mechanistic studies on the apparent association between parity and CCL rupture resistance. The research may also stimulate investigations into other non-reproductive health outcomes, such as autoimmune diseases, whose occurrence later in life may be impacted by parity.

**Abstract:**

Emerging evidence in women supports the notion that pregnancy may reset disease resistance, thereby providing protection against subsequent adverse health outcomes, but this hypothesis has not been adequately explored in domestic dogs. Cranial cruciate ligament (CCL) rupture is a degenerative orthopedic disease that frequently affects pet dogs, and its risk has been associated with disruption of the reproductive hormone axis. Our research team is conducting a lifetime cohort study of purebred Rottweilers in North America that have lived 30% longer than breed-average. Detailed medical and reproductive histories of 33 nulliparous and 32 parous Rottweilers were generated from questionnaires and review of medical records. Interviews with owners of bitches in the nulliparous group served to limit selection bias, confirming that in no instance was the reason for nulliparity based upon the owner’s suspicion that a bitch had a heightened risk for CCL rupture. The risk of CCL rupture associated with parity and other exposure variables was estimated using multivariate logistic regression. Overall, CCL rupture was diagnosed in 17 of 65 (26%) bitches. Median age at first litter and CCL rupture were 3.6 and 6.5 years, respectively. Compared to nulliparous, parous bitches had a significant 94% reduction in CCL rupture risk adjusted for duration of ovary exposure, overweight body condition, dietary pattern, habitual physical activity, and work/sport activity [OR_adjusted_ (95% CI) = 0.06 (0.01–0.46); (*p* = 0.006)]. The observed parity-associated CCL rupture risk reduction remained robust in sensitivity analysis excluding six nulliparous bitches for which decision not to breed was based on diagnosis of hip or elbow dysplasia, conditions which may be genetically linked to CCL rupture [OR_adjusted_ (95% CI) = 0.08 (0.01–0.58); (*p* = 0.01)]. This work sets the stage for replication studies in other canine populations that should begin to explore the mechanistic basis for parity-associated CCL rupture risk reduction and to pursue other non-reproductive health outcomes in bitches whose incidence or severity may be parity-sensitive.

## 1. Introduction

Pregnancy represents a significant deviation from normal physiology. Emerging evidence in women supports the possibility that pregnancy is a life event that may reset disease resistance, thereby lowering the risk of subsequent adverse health outcomes. In women, evidence is growing that breast cancer [1,2,3], dementia and rate of cognitive decline [4], as well as rate of aging [5], may be pregnancy-sensitive outcomes. Exploration of this paradigm in domestic dog populations has been largely limited to a few reports suggesting parous bitches may be less susceptible to pyometra than nulliparous bitches [6,7,8]. To our knowledge, the association between pregnancy or parity (i.e., the production of live offspring) and subsequent non-reproductive degenerative disease conditions in bitches has not been explored.

Once considered to be a consequence of trauma, it is now believed that the majority of cranial cruciate ligament (CCL) ruptures in dogs occur due to a degenerative, non-traumatic process—a progressive ligament fiber deterioration that may be accelerated by both local and systemic factors contributing to ligament failure [9,10]. Genetic and environmental factors both contribute to CCL rupture risk. Based upon studies of CCL rupture in Labrador Retrievers, a high-risk breed, CCL rupture is believed to be moderately heritable and highly polygenic [11].

Work by our group [12] and others [13,14] have reported a strong association between gonad exposure, age at gonad removal, and likelihood of CCL rupture, suggesting risk may be under endocrine influence. In Rottweilers, early endocrine disruption during the developmental period—removal of gonads during the first 24 months of life—was adversely associated with three indicators of susceptibility: increased incidence of CCL rupture; multiplicity (i.e., bilateral rupture); and earlier age at initial CCL rupture [12].

Our research team is conducting a lifetime cohort study of purebred Rottweilers living in North America that have lived 30% longer than breed average. The Exceptional Aging in Rottweilers Study (EARS) is an ambidirectional cohort study acquiring data prospectively and retrospectively from dogs reaching exceptional longevity, providing an opportunity to test the lifetime association between exposure variables and risk of CCL rupture unaffected by censoring because no dogs are lost to follow-up or die during the study period. We reasoned that, by combining detailed reproductive histories with rigorous review of lifetime medical records, this population might be ideally suited for the long-term evaluation of the association between parity and the lifetime occurrence of subsequent adverse health outcomes, such as veterinarian-diagnosed CCL rupture.

Based on the observed linkage between gonad status and lifetime cruciate ligament survival, we conducted an initial evaluation of the relationship between parity and CCL rupture in 26 female Rottweilers [12]. This work showed that among bitches that reached reproductive maturity (i.e., intact reproductive tract at 24 months of age), parous bitches had a significant 93% reduction in CCL rupture risk compared to nulliparous [12].

In this study, we extend those initial findings to evaluate an additional 65 Rottweiler bitches from the EARS study. The lifetime incidence of veterinarian-diagnosed CCL rupture in parous and nulliparous bitches were compared. To limit owner selection bias, standardized interviews with owners were used to confirm the reason for nulliparity was not based upon the owner’s suspicion of heightened risk of CCL rupture. In this report, we present data on reproductive history and lifetime CCL rupture diagnosis within the context of other exposure variables, including overweight body condition, dietary pattern, habitual physical activity, and work/sport activity, to address the research question: Is pregnancy and the resultant production of live offspring associated with significant protection or increased susceptibility to subsequent CCL rupture?

## 2. Materials and Methods

### 2.1. Study Population

The Exceptional Aging in Rottweilers Study (EARS) is an open registry, ambidirectional cohort study of client-owned male and female Rottweilers with exceptional longevity living in North America. Dogs enrolled in this study satisfy the following inclusion criteria: (1) Validation of purebred status through American Kennel Club (AKC) registry database; (2) age of >13 years, which represents living at least 30% longer than average lifespan of Rottweilers [15,16]; and (3) owner willingness to provide information by questionnaire, medical records, and telephone interviews to construct lifetime medical histories. As of 1 May 2024, 429 purebred Rottweilers with exceptional longevity have been enrolled.

Enrollment in EARS is owner-driven, initiated by owner contact with research staff in reference to a dog that may be study-eligible. Speaking engagements and interaction with veterinarians, dog owners, and dog breeders at national veterinary conferences and local and national breed club meetings were used to heighten awareness of the need for longevity research in pet dogs and to enhance study visibility. Enrollment of dogs living in North America is not limited to a particular geographic region, based upon proximity to investigators, or dependent upon residence in urban-suburban-rural settings. Dogs in the EARS study are not enrolled based upon their reproductive history or whether their medical history reveals that they have been affected by any particular medical condition, such as CCL rupture. In this study, pet owners completing questionnaires and telephone interviews were unaware of any CCL rupture-related inquiry because the stated research goal was to advance the scientific understanding of the determinants of longevity without targeting a particular disease.

**Figure 1:**
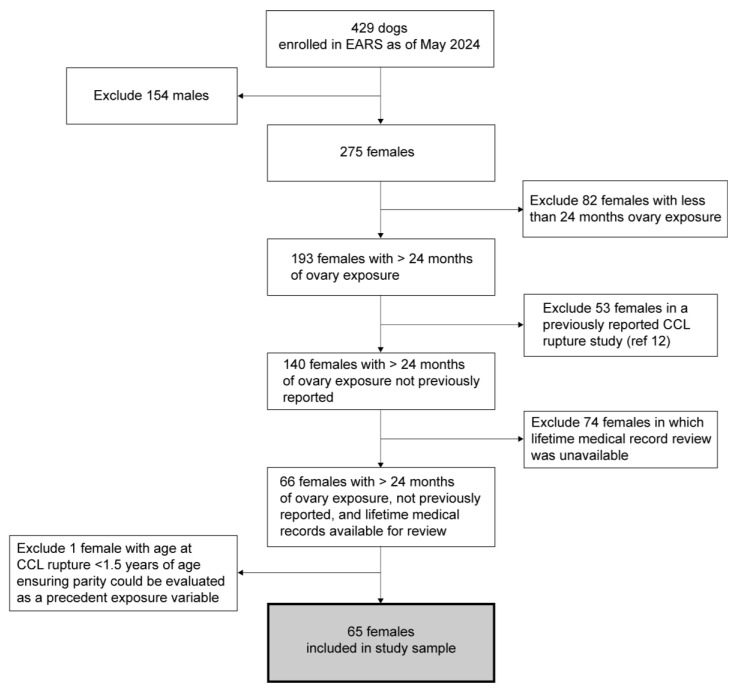
Flow chart of Rottweiler bitches included in the study sample after applying inclusion and exclusion criteria to the Exceptional Aging in Rottweilers Study (EARS) cohort (see Section 2.1).

This report utilizes data on a subset of Rottweilers enrolled in EARS. A total of 429 study subjects were initially available (Figure 1). For this analysis, we excluded (1) 154 males and (2) 82 females that had gonad removal during the first 24 months of life to eliminate females that did not reach reproductive maturity. Because our earlier analysis indicated that lifetime medical histories built upon questionnaires but lacking medical records review significantly underestimated the occurrence of CCL rupture (the primary outcome of this report), we excluded 74 bitches for which medical records review was not available. Fifty-three additional bitches were excluded from this research sample because they were included in a prior published study of CCL rupture [12], therefore none of the cases in this report were previously reported. Finally, we excluded bitches with CCL rupture diagnosis occurring at less than 1.5 years of age—the earliest age at first whelping among all parous females in the EARS study—which enabled us to evaluate parity as a *precedent exposure variable*, i.e., parity occurring prior to subsequent CCL rupture. This resulted in the exclusion of one bitch. Thus, the final analytical sample described in this report consists of 65 purebred Rottweiler bitches satisfying the following criteria: (1) at least 24 months of intact reproductive tract; (2) information on duration of ovary exposure, body condition, dietary pattern, habitual physical activity, and work/sport activity; (3) medical records available for review to assess lifetime incidence of veterinarian-diagnosed CCL rupture; (4) information on reproductive history to assign parous versus nulliparous, number of litters, age at first litter, age at last litter; and (5) completed interviews with owners of bitches in the nulliparous group to obtain reason for nulliparity.

### 2.2. Ascertainment of CCL Status

The diagnosis of CCL rupture was based upon an owner-completed questionnaire and a medical records review. In 12 of 17 bitches with CCL rupture, a diagnosis of ligament rupture occurred at the time of open stifle surgery. Non-surgical cases were diagnosed based on a clinical history of acute stifle lameness, excess cranial–caudal instability, or the finding of periarticular fibrosis and crepitus consistent with the chronic periarticular changes that accompany chronic CCL rupture.

Age at CCL rupture was obtained from questionnaire and medical records. For bitches that had their CCL rupture surgically repaired, age at rupture was considered to be the age at surgery, i.e., age at visual confirmation. For bitches that did not have their CCL surgically repaired, age at rupture was recorded as the age at the first recorded visit to a veterinary clinic with stifle lameness attributable to CCL injury. In one bitch, age at CCL rupture was not available. Time logs were constructed to visualize the temporal relationship between CCL rupture and age at whelping and between CCL rupture and age at ovariohysterectomy.

### 2.3. Reproductive History, Reason for Nulliparity

Questionnaire and telephone interview were used to collect detailed information on lifetime reproductive history for each bitch. Information on date at whelping, age at first litter, age at last litter, and number of litters was obtained. Based on reproductive history, bitches were categorized as: (1) parous (producing live offspring); or (2) nulliparous (no live offspring).

Information used to categorize the reason for nulliparity was collected from a standardized telephone interview with each owner of the 33 bitches in the nulliparous group. The following reasons were identified: (1) never had an intention to breed; (2) intention to breed but inadequate time/other commitments; (3) reproductive failure; (4) behavioral issues; (5 and 6) presence of hip dysplasia or elbow dysplasia detected on orthopedic screening; (7) non-orthopedic substandard conformation (e.g., dental malocclusion, hair color); and (8) other disease conditions, including subaortic stenosis and immune-mediated thrombocytopenia. This interview revealed whether any dogs had a reason for nulliparity that was a condition that could have influenced CCL rupture risk, such as hip dysplasia or elbow dysplasia [17]. Importantly, the information obtained from these interviews enabled us to determine how frequently an owner’s concern of heightened CCL rupture risk influenced the decision not to breed. If this concern frequently influenced the decision for nulliparity, it could introduce an owner selection bias that could spuriously lead to an increased frequency of CCL rupture occurring in the nulliparous group.

### 2.4. Other Exposure Variables

#### 2.4.1. Lifetime Ovary Exposure

Lifetime gonad exposure (in years) reflected age at gonad removal. In most cases, age at gonad removal was calculated by comparing the date of birth and the date of gonad removal surgery using medical records. When medical record validation of the date of gonad removal was unavailable, the gonad removal date provided in the questionnaire by the owner was used. If the date was recorded as month/year in the questionnaire, the 15th day of the particular month was recorded as the estimated age at gonad removal.

No bitches underwent gonad removal for the prevention or treatment of CCL rupture. In logistic regression models of CCL rupture risk, gonad exposure was handled as a dichotomous risk variable using median ovary exposure (5.8 years) as the cut-point.

#### 2.4.2. Body Condition

Owner-reported body condition of each bitch was collected retrospectively by questionnaire. Owners were asked to select one of four possible body conditions [underweight; ideal; overweight; markedly overweight (obese)] for three different periods during the life course [pre-adult (6–9 months of age); middle-aged adult (4–6 years of age); and older adult (more than 7 years)]. Owner-reported body condition assessments were temporally matched with age at CCL rupture so that bitches could be grouped as: (1) overweight prior to rupture of CCL (i.e., precedent overweight); or (2) not overweight prior to rupture of CCL.

Logistic regression was used to estimate the risk of CCL rupture associated with being overweight prior to rupture of CCL. Each owner could only report one body condition assessment per life course period. Because a bitch’s body condition could change during the life course period in which CCL rupture diagnosis occurred, data were handled to maximize the number of bitches coded as precedent overweight. This maximized the likelihood of detecting an association between precedent overweight and CCL rupture risk. Among bitches with bilateral CCL rupture, none of the bitches with ideal body condition prior to first rupture became overweight prior to second rupture.

#### 2.4.3. Dietary Pattern

Consumption of a raw meat diet is a canine dietary pattern that may be readily differentiated from other dietary patterns using information provided by owners. Using questionnaire-based owner reports of diet consumed by bitches from the age of 9 months to 13 years, each bitch was categorized with respect to their raw diet consumption as: (1) never; (2) sporadic; or (3) daily. Daily raw diet was assigned to bitches that consumed this dietary pattern daily (with no consumption of commercial dog food) for at least 10 years. For logistic regression models, bitches with sporadic or daily intake were combined so that raw diet consumption was dichotomized as “ever” versus “never”.

#### 2.4.4. Habitual Physical Activity

Owner-reported physical activity of bitches was collected retrospectively by questionnaire. For three physical activity categories—walking, swimming, running/jogging—owners selected one of four possible frequencies: never; daily; weekly; or monthly. In each instance, owners were asked to provide information on the pattern of physical activity when the bitches were middle-aged adults (i.e., 4–6 years old). Walking was defined as a distance of at least 100 yards; swimming was defined as swimming for a duration of 30 min; running/jogging was defined as a duration of at least 30 min. These responses were used to construct an integrated habitual physical activity profile that may reduce orthopedic risk consisting of daily walking, any swimming, and no running or jogging. For logistic regression, habitual physical activity was dichotomized as “low risk” versus “high risk”. Low-risk bitches had responses consistent with the reduced-risk activity profile in at least two of the three physical activity categories (i.e., daily walking, swimming, or absence of running/jogging). High-risk bitches had zero to one reduced risk response within the three physical activity categories.

#### 2.4.5. Participation in Work/Sport Activities

Owner-reported participation of bitches in work/sport activity was collected retrospectively by questionnaire and by telephone interview. Work/sport activities included: agility; barn hunt; carting; endurance; herding; nosework; obedience; rally; tracking, and traffic. Conformation, good citizenship, temperament, and therapy were not considered as categories of work/sport activity in this analysis. In logistic regression, participation in work/sport activity was treated as a dichotomous variable: “no work/sport activity” (no title in a work/sport activity) versus “work/sport activity” (title from one or more of the work/sport activity categories listed above). For each activity, no discrimination was made between titles reflecting a lower or higher level of achievement or differences in geographic site or frequency of competition.

#### 2.4.6. Adult Height

Adult height measured from the floor to the top of the shoulder (withers) was reported by owners. For logistic regression, owner-reported adult height was dichotomized (tall versus short) and also categorized as tertiles (short, medium, tall). Adult height was not included in the overall multivariable model of CCL rupture risk because 15 bitches had missing values. Instead, a separate logistic regression of data from 50 bitches was used to evaluate the relationship between adult height and CCL rupture. The extent to which differences in adult height attenuated the observed association between parity and CCL rupture risk reduction was also determined.

### 2.5. Statistical Analysis

Data analyses were performed using SPSS version 29 [18]. Statistical significance was set at *p* < 0.05; all tests were two-sided. Nulliparous and parous bitches were compared with respect to age at death, duration of ovary exposure, adult height, overweight body condition, raw diet consumption, habitual physical activity, and participation in work/sport activity. For nulliparous and parous groups, variables were expressed as medians [interquartile range (IQR), range] or proportions (%) and compared using either chi-square test for association, Fisher’s exact test, or Mann–Whitney U test. For the calculation of CCL rupture incidence rates, the number of dog years at risk (DYAR) was based on the 13-year study observation period and time to the first CCL rupture. To be consistent with previously published estimates of CCL rupture incidence in Rottweilers [12,19], incidence rates in this study sample were expressed as cases of CCL rupture per 10,000 DYAR.

To estimate the risk of CCL rupture associated with parity and other exposure variables, a logistic regression model was used to generate odds ratios (OR). Chi-square test for association was used to assess multicollinearity between categorical independent variables. To assess each association, a phi (φ) value was calculated [20]. For our inferential testing, we considered φ values less than 0.7 as manageable [21]. We found modest interrelationships between covariates, with the only significant association being between parity and duration of ovary exposure (φ = 0.48, *p* < 0.001). Risk variable selection in final models was guided by practical considerations to achieve inferential models rather than focusing on the elimination of variables to create parsimonious predictive models. No censoring prior to the end of the study observation period occurred since no bitches died or were lost to follow-up between 0 and 13.0 years of age. To test the association between parity and CCL rupture risk, non-adjusted and adjusted (ovary exposure, body condition, raw diet consumption, habitual physical activity, work/sport activity) ORs and 95% confidence intervals (95%CI) were reported. In these analyses, the nulliparous group was assigned as the reference group (OR = 1.0) so that odds ratios of the parous group less than 1.0 reflected an estimate of risk reduction.

To test the robustness of our main finding of parity-associated CCL rupture risk reduction, a sensitivity analysis was performed by constructing a logistic regression model for 59 bitches after excluding six nulliparous bitches in whom owner-reported reason for nulliparity was the diagnosis of another condition based on orthopedic screening (hip dysplasia or elbow dysplasia) that may have a genetic linkage with CCL rupture risk [17]. If the bitches excluded in this sensitivity analysis were enriched for CCL rupture risk alleles and contributed significantly to the elevated CCL rupture risk in the nulliparous group, their exclusion would be hypothesized to strongly attenuate the association between parity and CCL rupture risk reduction observed in the overall study sample.

## 3. Results

### 3.1. Reproductive History and CCL Rupture Incidence in the Overall Study Sample

This study sample consisted of 65 purebred female Rottweilers that lived at least 13 years and were reproductively intact during the first two years of life. Median (IQR) age at death was 14.0 (13.6–14.5) years. Thirty-two of 65 (49%) bitches were parous. The number of litters ranged from 1 to 4 (median = 2 litters). Median (IQR) age at first litter was 3.6 (2.8–4.2) years. Among multiparous bitches, median age at last litter was 6.4 years (range, 2.9–8.7 years). Median (IQR) age at ovariohysterectomy (spay) was 5.8 (3.9–7.1) years. The temporal relationship between ovariohysterectomy and CCL rupture varied broadly; on average, ovariohysterectomy occurred 1.3 years prior to CCL rupture, ranging from ovariohysterectomy at 9.7 years prior to CCL rupture to the procedure occurring 2.9 years after CCL rupture. No bitches underwent genetic screening to assess CCL rupture risk. No bitches underwent ovariohysterectomy with the intent of treating or preventing CCL rupture.

Overall, 17 of 65 (26%) bitches had a lifetime diagnosis of CCL rupture (Table 1). In 12 of these bitches, CCL rupture was confirmed at the time of open stifle surgery. Median age at diagnosis of first CCL rupture was 6.5 years (range, 2.6–12.5 years). Bilateral CCL rupture was diagnosed in 10 of 17 (59%) affected bitches, with a median interval to second rupture of 25 months (range, 4–60 months). CCL rupture incidence rate was 237 cases per 10,000 dog-years-at-risk.

### 3.2. Comparison of Characteristics of Bitches in the Nulliparous and Parous Groups

**Figure 2:**
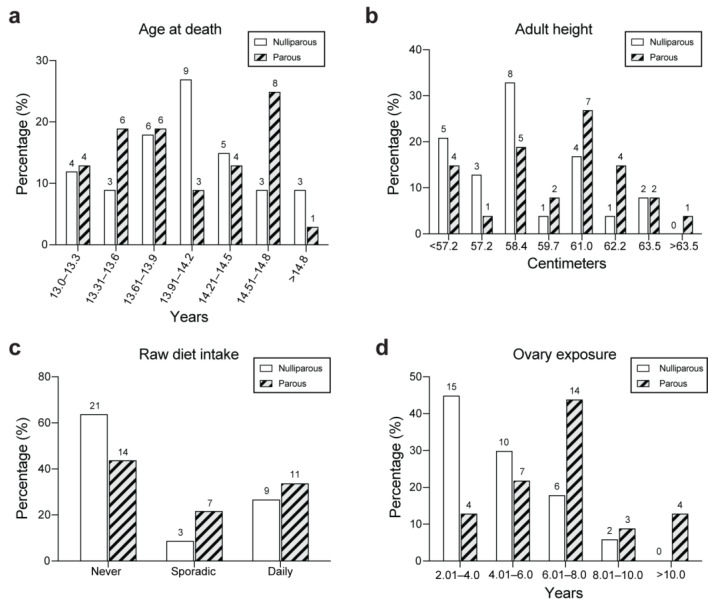
Histograms showing the frequency distribution of age at death (**a**), adult height (**b**), raw diet intake (**c**), and duration of ovary exposure (**d**) in 33 nulliparous and 32 parous bitches. Number above bar = number of bitches. Percentage reflects the proportion of bitches within the nulliparous or parous group.

A descriptive comparison of characteristics of the 33 nulliparous and 32 parous females in this study cohort is presented in Table 1. Figure 2 (panels a–d) shows frequency distribution of age at death, adult height, raw diet intake, and age at ovariohysterectomy in nulliparous and parous bitches. Parous and nulliparous bitches did not differ with respect to age at death, adult height, and the proportion of dogs that had overweight body condition. Parous and nulliparous bitches did not differ in habitual physical activity or participation in work/sport activity. Parous bitches had a significantly greater number of years of being reproductively intact (median, 6.8 years) compared to nulliparous (median, 4.3 years) (*p* < 0.001). The proportion of bitches that consumed raw diet was higher in the parous group (56%) than in the nulliparous group (36%), but the difference did not reach statistical significance (*p* = 0.11).

### 3.3. Parity, the Production of Live Offspring, Is Associated with Significant CCL Rupture Risk Reduction

CCL rupture was diagnosed in 14 of 33 (42%) nulliparous bitches. In contrast, only 3 of 32 (9%) parous bitches had CCL rupture. Compared to nulliparous bitches, parous bitches had a significant 86% reduction in the risk of CCL rupture [OR (95% CI) = 0.14 (0.04–0.56); (*p* = 0.005)] P (Table 2). The strength of this parity-associated CCL rupture risk reduction remained strong (94% reduction) in multivariable analysis adjusting for age at ovariohysterectomy, overweight body condition, dietary pattern, habitual physical activity, and work/sport activity [OR_adjusted_ (95% CI) = 0.06 (0.01–0.46); (*p* = 0.006)] (Table 2). The CCL rupture incidence rate was 5.5-fold lower among parous bitches compared to nulliparous; incidence rates were 77 cases versus 427 cases per 10,000 DYAR, respectively (*p* = 0.003) (Table 1). A greater number of litters was not associated with stronger rupture risk reduction or increased rupture risk; 14 bitches with one litter and 18 bitches with two or more litters did not differ significantly in their likelihood of CCL rupture diagnosis (*p* = 0.70).

An increase in the likelihood of CCL rupture was associated with participation in work/sport activity [OR_adjusted_ (95% CI) = 5.43 (1.27–23.51); (*p* = 0.02)]. (Table 2). Bitches segregated into low-risk versus higher-risk habitual physical activity did not differ in their likelihood of CCL rupture. Neither bitches that consumed a raw diet nor bitches that had a precedent overweight body condition had an increased likelihood of CCL rupture (Table 2). Owner-reported tall adult height was not significantly associated with CCL rupture risk [OR (95% CI) = 0.72 (0.19–2.65); (*p* = 0.62)]. Accounting for differences in adult height did not attenuate the association between parity and CCL rupture risk reduction.

### 3.4. The Main Finding, the Association between Parity and CCL Rupture Risk Reduction, Is Not Explained by Owner Decision on Why Nulliparous Females Did Not Become Parous

**Figure 3:**
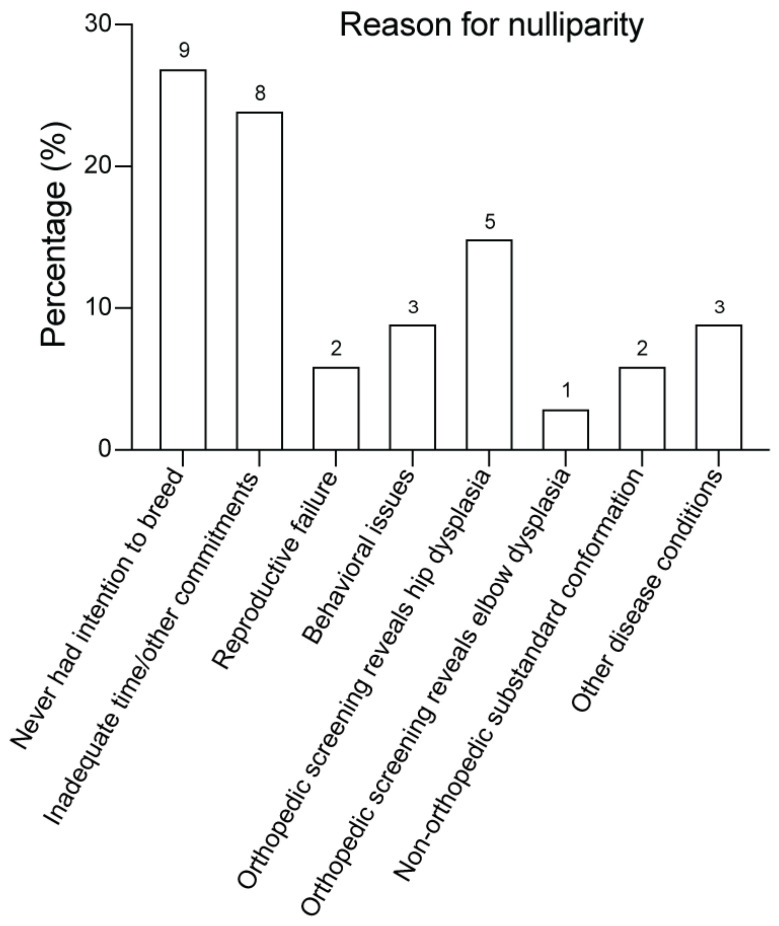
Histogram showing reason for nulliparity among the 33 bitches in the nulliparous group. Information to categorize reason for nulliparity was collected by conducting a standardized telephone interview with each owner. Eight categories of reason for nulliparity are shown. Interviews with owners confirmed that in no instances was reason for nulliparity based upon the owner’s suspicion that a bitch had heightened risk for cranial cruciate ligament (CCL) rupture. Number above bar = number of bitches. Percentage reflects the proportion of bitches in the nulliparous group.

To address a potential selection bias that could account for the higher frequency of CCL rupture observed in the nulliparous group, owners of nulliparous bitches underwent a detailed interview to establish their reason for choosing nulliparity. In none of the bitches was the decision for nulliparity based upon an owner’s suspicion that a particular bitch may be at heightened risk for CCL rupture that could be passed to offspring. The eight categories of reason for nulliparity are shown in Figure 3. The most common reason for nulliparity was “never had an intention to breed” (*n* = 9), followed by “intention to breed but inadequate time/other commitments” (*n* = 8). Reproductive failure was reported in only two nulliparous bitches. Artificial insemination (twice in one bitch, four times in another) did not result in pregnancy; neither bitch with reproductive failure had a lifetime diagnosis of CCL rupture.

In six nulliparous bitches, orthopedic screening revealed hip dysplasia (*n* = 5) or elbow dysplasia (*n* = 1). In these bitches, the owner’s decision not to breed was based upon the intention to avoid hip dysplasia or elbow dysplasia from being passed to offspring. Because canine hip dysplasia, elbow dysplasia, and CCL rupture may share risk alleles [17], a re-analysis of CCL rupture risk was conducted after exclusion of these six nulliparous bitches. In this sensitivity analysis, there was a 92% protective association between parity and CCL rupture risk [OR_adjusted_ (95% CI) = 0.08 (0.01–0.58); (*p* = 0.01)], supporting the robustness of the strong parity-associated CCL rupture risk reduction found in our primary analysis.

## 4. Discussion

The intriguing possibility that pregnancy represents a life event capable of influencing disease resistance, thereby altering the risk of females developing subsequent non-reproductive health outcomes, prompted this inquiry. While data are emerging to support this notion in women, the hypothesis has not been adequately explored in domestic dog populations. Here, we constructed detailed lifetime reproductive and medical histories in a cohort of long-lived purebred Rottweilers, enabling a long-term evaluation of parity, as a precedent exposure variable, with the lifetime occurrence of CCL rupture, a canine degenerative orthopedic disease. In this lifetime study of the relationship between parity and subsequent CCL rupture, parous bitches had a significant 86% reduction in CCL rupture risk compared to nulliparous bitches, and this effect size was not attenuated when covariates were included in multivariable regression models. In order to eliminate a critical potential selection bias, owner interviews were utilized to confirm that in none of the nulliparous bitches was the decision for nulliparity based upon the owner’s concern for heightened risk of CCL rupture. Moreover, the 5.5-fold lower CCL rupture incidence rate among parous females could not be explained by differences between parous and nulliparous groups with respect to other exposure variables, including lifetime ovary exposure, overweight body condition, raw diet consumption, habitual physical activity, and participation in work/sport activity, which might have influenced CCL rupture risk. Further support for the apparent parity-associated protection against CCL rupture was provided by sensitivity analysis excluding nulliparous bitches with a screening diagnosis of hip or elbow dysplasia, conditions which may have genetic linkage with CCL rupture risk [17]. The findings reported here offer justification for mechanistic studies to further evaluate the association between parity and CCL rupture in bitches. Moreover, these results provide the rationale for future canine studies to evaluate other non-reproductive health outcomes whose prevalence, severity, or rate of progression may be parity-sensitive. By re-envisioning the possibility that an organism-wide spectrum of biological trade-offs can be induced by pregnancy, this new line of inquiry could redirect research emphasis away from solely studying external factors and physiological changes that jeopardize or promote healthy pregnancy to move toward understanding the potential impact of parity on lifetime health.

The results presented here should be considered preliminary and hypothesis-generating. Yet the data pattern fits squarely with an emerging paradigm in women, untested in bitches, that pregnancy can significantly influence the susceptibility to subsequent non-reproductive disease outcomes. There is growing scientific and practical interest in elucidating these linkages. Data from women show that pregnancy may either confer *disease protection* (e.g., cognition [4], all-cause mortality [22]) or *heighten disease risk* (e.g., abdominal obesity [23], hypertension [24], cardiovascular disease [25]). This bidirectionality of influence is best illustrated by the epidemiology of breast cancer in women, where early pregnancy (20–25 years old) is associated with breast cancer risk reduction, whereas pregnancy after 35 years is associated with *increased* breast cancer risk [1,26,27,28]. The evidence accumulating in women that parity may exert a long-standing impact on subsequent health outcomes suggests these effects are not solely attributable to temporary changes in gestational hormones but rather a mechanism that is durable. The epigenetic reprogramming of gene expression in a wide array of target tissues provoked by pregnancy [3,29,30,31,32,33] provides a dynamic yet durable long-term memory that can persist long after the provoking stimulus is no longer present. The pursuit of epidemiological linkages between pregnancy and subsequent health outcomes in bitches would appear to be an area ripe for investigation. Focus on the transcriptional signatures of immune cells and connective tissues, including neuromuscular units, may offer prime targets for defining the mechanisms underlying a possible parity-induced epigenetic memory.

Contemporary thought envisions the vast majority of cruciate ligament ruptures in dogs as the consequence of a degenerative process, not traumatic injury [9,10]. Both genetic and non-genetic factors contribute to canine CCL rupture risk. Detailed genetic studies in Labrador Retrievers suggest the risk of CCL rupture is a highly polygenic, moderately heritable trait [11]. Genomic studies linking genetic loci with canine CCL rupture risk implicate numerous contributing pathways, including regulators of inflammation and immune response [11,34], connective tissue and extracellular matrix [35], and neuromuscular integrity [36]. Studies from women and rodents now provide evidence that pregnancy can epigenetically reprogram functional pathways—biological pathways that mirror those linked to the CCL rupture risk variants implicated by the canine genetic studies—resulting in gene silencing and durable associations with changes in disease risk [29,33]. For example, pregnancy with the production of live offspring has been shown to be associated with a reduction in long-term disability progression in women with multiple sclerosis [37]. In multiple sclerosis patients, investigators have described a parity-induced signature of methylation changes in peripheral immune cells that persists for at least 16 years after pregnancy that may favorably influence neural plasticity and patient outcome [37].

If pregnancy leaves residual influences on inflammatory markers [38], it is reasonable to postulate that parity-provoked alterations in immunologic and/or inflammatory trajectories over the life course may significantly impact the likelihood of developing a degenerative disease such as canine CCL rupture, which is preceded by synovial inflammation [39]. Here, in purebred female Rottweilers, a breed at elevated risk of CCL rupture, we show an association between the production of live offspring and a reduction in veterinarian-diagnosed CCL rupture that persists over a 13-year life span, a duration extending almost 10 years after the median age at first whelping. Further, we show no increased protection with an increasing number of litters, consistent with an epigenetic reset hypothesis whose long-term memory does not rely upon multiple pregnancy events [22,40].

To explore alternative explanations for the occurrence of more CCL rupture cases among nulliparous bitches in this study sample, we closely examined characteristics that differed between the nulliparous and parous groups. Of utmost importance, we examined the possibility that selection bias related to owner perception or knowledge of increased CCL rupture susceptibility may have resulted in bitches landing in the nulliparous group. After conducting reason-for-not-breeding interviews with owners of the 33 bitches in the nulliparous group, we found no evidence that owner-suspected heightened CCL rupture risk influenced the decision not to breed in any of the bitches in this study sample. Considering the possibility that the screening diagnosis of hip dysplasia or elbow dysplasia—which was reported by six owners as the reason for nulliparity—might increase the likelihood of CCL rupture, we performed a sensitivity analysis removing these six bitches from the nulliparous group and documented there was only slight attenuation of the observed parity-associated CCL rupture risk reduction.

Parous and nulliparous groups differed in their lifetime duration of ovary exposure. Ovary exposure in the parous group exceeded by 2.5 years the average ovary exposure in the nulliparous group, raising the possibility that earlier ovary removal in the nulliparous bitches might have contributed to the increased occurrence of CCL rupture. In our study sample, we found a linkage between shorter ovary exposure and increased CCL rupture risk in univariate analysis, but the association was no longer significant when parity and other exposure variables were accounted for. In a separate analysis of only the nulliparous females in this study sample, shorter ovary exposure after 24 months was not associated with an increased risk of CCL rupture. In previous work, we showed that early endocrine disruption during the first 24 months of life in Rottweilers was associated with adverse CCL outcomes, reflected by increased incidence of CCL rupture, increased occurrence of bilateral rupture, and earlier age at first rupture [12]. In that study, we also documented that the decision to remove or retain gonads between the ages of 2–4 years, 4–6 years, and 6–8 years had no bearing on CCL rupture risk. From these results, we posited there may be a sensitive gonad exposure window during the developmental period that can jeopardize lifetime cruciate ligament survival in Rottweilers and some other breeds, a notion consistent with data on the timing of gonadectomy reported by Hart and colleagues [13,14,41,42]. The current study does not provide additional evidence to support or refute the relationship between gonad exposure during development and subsequent CCL rupture because none of the bitches in this study sample underwent gonad removal during the 24-month developmental period.

Consumption of raw diet was more common among parous females than nulliparous females (56% versus 36%, respectively). This observation prompted us to interrogate the relationship between raw diet, parity, and CCL rupture. If parity is segregated with raw diet consumption and raw diet consumption is associated with CCL rupture protection, then the strong association between parity and CCL rupture risk reduction might be explained in part by the difference in dietary pattern observed in the parous and nulliparous groups. However, consumption of raw diet was not associated with CCL rupture risk reduction. We found the opposite trend. Overall, bitches that had sporadic or daily intake of raw diet had a 1.4-fold *increase* in the likelihood of CCL rupture compared to bitches that never consumed raw diet. This association strengthened to a 2.4-fold increased risk in multivariable analysis. Thus, we found no evidence that the observed association between parity and risk of CCL rupture in this study sample could be attributed to differences in raw diet consumption. Future efforts should seek to further evaluate the possible role of dietary pattern in the pathogenesis of CCL rupture.

Investigators have proposed that being overweight or obese may contribute to the risk of canine CCL rupture, possibly mediating the relationship between early gonadectomy and an increase in CCL rupture [43,44,45]. In the current study sample of bitches that did not undergo gonadectomy during the first 24 months of life, we found no significant difference in the prevalence of owner-reported overweight body condition between parous and nulliparous bitches. Also, we found that being overweight prior to CCL rupture was not a significant precedent risk variable in our models of CCL rupture risk, consistent with our earlier findings on lifetime cruciate ligament survival in Rottweilers [12]. It should be noted that, rather than using veterinarian-reported body condition scores, we relied upon owner-reported body condition. This may have resulted in misclassification or underestimation of the number of bitches that were overweight, thereby contributing to null associations. We conclude that our findings reported here fail to support the notion that differences in the occurrence of overweight body conditions were a major underlying determinant of our main finding, namely the association between parity and CCL rupture risk reduction.

In this study sample, we found no significant association between owner-reported adult height and CCL rupture. Previously, we reported that tall height in Rottweilers was associated with an increased likelihood of veterinarian-diagnosed CCL rupture [12]. There are several potential explanations for this apparent inconsistency. In the previous report, adult height did not rely upon owner reports but instead relied upon precise, repeated shoulder height measurements performed by a single veterinary examiner. In that report, the association between tall adult height and the risk of CCL rupture was stronger among males than females, and the study cohort included Rottweilers that underwent gonad removal during the 24-month developmental period, which may have resulted in delay in growth plate closure with subsequent prolongation of longitudinal bone growth [46,47,48]. In contrast, the present study evaluated only females that were reproductively intact during the first 24 months of life. The taller height observed among parous females in the present study sample might have been expected to favor an increase in the risk of CCL rupture in the parous group. It did not. Instead, parous bitches had a 5.5-fold lower CCL rupture incidence rate. Future breed-specific studies using standardized measurements of height should explore factors that moderate the relationship between adult height and risk of canine CCL rupture.

We evaluated the likelihood that differences in habitual physical activity were associated with differences in risk of CCL rupture. Our results indicate: (1) Habitual physical activity was not a risk factor for lifetime diagnosis of CCL rupture; (2) habitual activity was not different between females in the parous and nulliparous groups; and (3) inclusion of habitual activity as an exposure variable in our multivariable models of CCL rupture risk did not attenuate the association between parity and CCL rupture risk reduction. These null findings are consistent with results from 412 purebred Labrador Retrievers reported by Terhaar et al. [49], who showed that questionnaire-based habitual activity patterns were not significantly different between dogs with CCL rupture versus controls. In our study, low-risk and high-risk physical activity patterns were also generated using owner questionnaire responses, raising the possibility that null results might not accurately reflect the real impact of habitual physical activity on the likelihood of CCL rupture, either because of difficulty in owner recall or the inadequacy of the collected responses to capture the environmental aspects of physical activity critical to CCL rupture risk.

We evaluated owner-reported participation of bitches in 10 different work/sport activities. We found no difference in owner-reported participation in work/sport activities between the parous and nulliparous groups. There was, however, a more than 5-fold higher likelihood of CCL rupture in bitches participating in at least one work/sport activity compared to those bitches that did not compete, an association that remained significant in multivariable analysis. The most detailed published study on the relationship between work/sport activity and diagnosis of CCL rupture was reported by Sellon and Marcellin–Little [50]. Their study focused on a particular activity, agility, using questionnaire-based data from more than 1200 dogs and showed the risk of CCL rupture was increased in some competitors (novice and intermediate levels, fewer than 10 events per year), whereas CCL rupture occurrence was decreased in competitors at higher levels and those who engaged in other work/sport activities, such as nosework and barn hunt. Our data collection and analysis did not discriminate between particular categories of work/sport activity, competition at different levels, or frequency of competition. Clearly, our exposure variable work/sport activity represents a heterogeneous collection of physical demands and degrees of athletic conditioning and training. Further research into the linkage between training regimens, particular work/sport activities, and an increase or decrease in CCL rupture risk is justified.

Our study has limitations. Much of the data in this lifetime cohort study were collected retrospectively, which may limit its accuracy and precision. However, reproductive events are memorable to owners and breeders. Therefore, we suspect the categorization of bitches as either nulliparous or parous is highly accurate. Indeed, the dates of whelping were available for 28 of 32 parous females, providing a high level of confidence in the reproductive status of this study population. We defined parity as a litter with live offspring, therefore we did not account for pregnancies that yielded no live births. Although it is unlikely that this decision would have changed the status of many females from nulliparous to parous, the recorded number of pregnancies and age at first gestation would have changed in some bitches. Lactation, gestation length [2], and complications of pregnancy were not considered. We had insufficient number of CCL rupture cases among parous bitches to adequately explore linear versus U-shaped dose responses [51] between the number of litters and CCL rupture risk, or whether earlier age at first litter conferred greater protection. The key exposure variable, pregnancy with resultant live offspring, was not randomized among bitches in this cohort, and therefore causality cannot be assigned to the observed associations. The incidence of veterinarian-diagnosed CCL rupture relied upon lifetime medical health questionnaires completed by the owner and medical records reviewed by research personnel. However, underdetection of CCL rupture in this study sample, which is likely to have occurred due to lack of standardized late-life physical or radiographic examination [12], is unlikely to have been differential based upon parous versus nulliparous reproductive status.

This cohort of North American Rottweiler bitches that reached exceptional longevity represents a unique population. Therefore, the associations and effect sizes reported here may not directly translate to other Rottweiler populations or other breeds. However, this unique cohort offers several potential advantages, enabling a rich description of exposure variables and lifetime incidence of veterinarian-diagnosed CCL rupture, providing a setting well-suited for assessing the durability of risk reduction throughout the life course. Importantly, this work sets the stage for replication studies in other canine populations that should begin to explore the mechanistic basis for the apparent association between parity and protection against CCL rupture. Considered more broadly, the work opens the door to pursuing other non-reproductive health outcomes in bitches whose incidence or severity may be parity-sensitive.

Finally, several aspects of this study design may be considered strengths, including: focus on a single breed; confirmed purebred status; lifetime medical histories of North American bitches that did not undergo spaying prior to reproductive age; reliable classification of reproductive status (nulliparous versus parous); parity evaluated as a precedent exposure variable; sensitivity analysis to heighten confidence in the robustness of the main findings of the primary analysis. Importantly, the study utilized standardized interviews with owners that secured detailed information on reason-for-not-breeding to examine closely the likelihood that owner concern with heightened CCL rupture risk may have resulted in nulliparity, thereby eliminating a critical potential selection bias.

## 5. Conclusions

To explore an intriguing hypothesis—that pregnancy represents a life event that may enhance or diminish susceptibility to subsequent non-reproductive health outcomes—we collected data on reproductive histories and the lifetime occurrence of a degenerative orthopedic condition, cranial cruciate ligament (CCL) rupture, in purebred Rottweiler bitches to demonstrate that parous bitches had significantly lower CCL rupture risk compared to nulliparous. The observed 5.5-fold lower CCL rupture incidence among parous females could not be explained by parous-nulliparous group differences in other exposure variables encompassing diet, body condition, and physical activity, nor could it be explained by owner selection bias that could have favored bitches with a heightened risk of CCL rupture landing in the nulliparous group. These findings support the results of our earlier report [12] and set the stage for replication studies in other dog populations that should advance the mechanistic basis for a possible nexus between parity, susceptibility to CCL rupture, and the risk of other non-reproductive health outcomes.

## Figures and Tables

**Table 1:** Characteristics of 65 bitches from the Exceptional Aging in Rottweilers Study (EARS). For the exposure variables of birth cohort, ever overweight, raw diet, habitual physical activity, and work/sport activity, nulliparous and parous groups were compared using a Chi-square test for association. For age at death, ovary exposure, adult height, and age at first rupture, nulliparous and parous groups were compared using the Mann–Whitney U test. Differences in CCL status between nulliparous and parous bitches were assessed using Fisher’s exact test. ^a^ Significant difference between nulliparous and parous groups. ^b^ Owner reported height was available for 50 bitches. CCL = cranial cruciate ligament; # = number; IQR = interquartile range; DYAR = dog-years at risk.

Variable	Females from the Exceptional Aging in Rottweilers Study			
	Total (*n* = 65)	Nulliparous (*n* = 33)	Parous (*n* = 32)	*p*-Value
**Residence**				
# of U.S. states, *n*	30 states and Canada	17 states and Canada	20 states and Canada	--
# of households, *n*	61	33	31	--
**Birth Cohort**				
1985–1997, *n* (%)	23 (35.4)	10 (30.3)	13 (40.6)	0.38
1998–2010, *n* (%)	42 (64.6)	23 (69.7)	19 (59.4)	
**Age at Death** (years), median (IQR)	14.0 (13.6, 14.5)	14.0 (13.7, 14.4)	13.9 (13.5, 14.7)	0.79
**Ovary Exposure** (years), median (IQR)	5.8 (3.9, 7.1)	4.3 (3.2, 5.9)	6.8 (5.6, 8.0)	<0.001 ^a^
**Adult Height** (cm), median (IQR) ^b^	58.4 (57.2, 61.0)	58.4 (57.2, 60.8)	61.0 (58.4, 62.2)	0.07
**Ever Overweight**, *n* (%)	22 (33.8)	10 (30.3)	12 (37.5)	0.54
**Raw Diet**				
Never, *n* (%)	35 (53.8)	21 (63.6)	14 (43.8)	0.11
Ever, *n* (%)	30 (46.2)	12 (36.4)	18 (56.3)	
**Habitual Physical Activity**				
Low risk, *n* (%)	44 (67.7)	20 (60.6)	24 (75.0)	0.22
High risk, *n* (%)	21 (32.3)	13 (39.4)	8 (25.0)	
**Work/Sport Activity**				
No work/sport activity, *n* (%)	37 (56.9)	18 (54.5)	19 (59.4)	0.69
Work/sport activity, *n* (%)	28 (43.1)	15 (45.5)	13 (40.6)	
**CCL Status**				
Any CCL rupture, *n* (%)	17 (26.2)	14 (42.4)	3 (9.4)	0.004 ^a^
Bilateral CCL rupture, *n* (% of CCL ruptures)	10 (58.8)	9 (64.3)	1 (33.3)	0.005 ^a^
Age at first rupture (years), median (IQR)	6.5 (4.9, 8.2)	5.8 (4.4, 8.4)	7.9 (7.7, n/a)	0.19
CCL rupture incidence rate (95% CI) expressed as cases per 10,000 DYAR	237 (138–380)	427 (233–716)	77 (16–226)	0.003 ^a^

**Table 2:** Unadjusted and adjusted odds ratios (OR) generated from logistic regression to estimate the risk of cranial cruciate ligament (CCL) rupture associated with parity and other exposure variables in nulliparous (*n* = 33) and parous (*n* = 32) bitches. For each variable, adjusted OR includes the other five variables in the multivariate analysis. Ovary exposure was dichotomized into two categories based upon median (5.8 years) ovary exposure. Habitual physical activity was dichotomized into low risk versus high risk based upon information on three categories of physical activity (see Section 2.4.4). OR (95% CI) = Odds Ratio and 95% confidence interval. ref = reference group.

Variable	Unadjusted OR (95% CI)	*p*-Value	Adjusted OR (95% CI)	*p*-Value
**Parity**				
Non-parous	1.0 (ref)	--	--	--
Parous	0.14 (0.04–0.56)	0.005	0.06 (0.01–0.46)	0.006
**Duration of Ovary Exposure**				
≥5.8 years	1.0 (ref)	--	--	--
<5.8 years	2.36 (0.75–7.42)	0.14	0.97 (0.21–4.60)	0.97
**Body Condition**				
Precedent Overweight	1.0 (ref)	--	--	--
Not overweight	1.34 (0.37–4.82)	0.66	0.52 (0.11–2.47)	0.41
**Raw Diet Intake**				
Never	1.0 (ref)	--	--	--
Ever	1.45 (0.48–4.39)	0.52	2.38 (0.60–9.47)	0.22
**Habitual Physical Activity**				
Low risk	1.0 (ref)	--	--	--
High risk	0.83 (0.25–2.78)	0.77	0.34 (0.07–1.58)	0.17
**Work/Sport Activity**				
No work/sport activity	1.0 (ref)	--	--	--
Work/sport activity	3.34 (1.05–10.64)	0.04	5.43 (1.27–23.51)	0.02

## Data Availability

The original contributions presented in this study are included in the article. Further inquiries or requests regarding the data can be directed to the corresponding author.

## Abbreviations

CCL, cranial cruciate ligament; DYAR, dog-years at risk; EARS, The Exceptional Aging in Rottweilers Study.
